# Dietary intake of animal-based products and likelihood of follicular lymphoma and survival: A population-based family case-control study

**DOI:** 10.3389/fnut.2022.1048301

**Published:** 2023-01-04

**Authors:** Michael K. Odutola, Marina T. van Leeuwen, Julie K. Bassett, Fiona Bruinsma, Jennifer Turner, John F. Seymour, Henry Miles Prince, Samuel T. Milliken, Mark Hertzberg, Fernando Roncolato, Stephen S. Opat, Robert Lindeman, Campbell Tiley, Judith Trotman, Emma Verner, Michael Harvey, Craig R. Underhill, Geza Benke, Graham G. Giles, Claire M. Vajdic

**Affiliations:** ^1^Centre for Big Data Research in Health, University of New South Wales, Sydney, NSW, Australia; ^2^Cancer Epidemiology Division, Cancer Council Victoria, Centre for Epidemiology and Biostatistics, Melbourne School of Population and Global Health, University of Melbourne, Parkville, VIC, Australia; ^3^Douglass Hanly Moir Pathology, Macquarie Park, NSW, Australia; ^4^Department of Clinical Medicine, Faculty of Medicine, Health and Human Science, Macquarie University, Sydney, NSW, Australia; ^5^Royal Melbourne Hospital, Peter MacCallum Cancer Centre, University of Melbourne, Melbourne, VIC, Australia; ^6^Epworth Healthcare and Sir Peter MacCallum Department of Oncology, University of Melbourne, Parkville, VIC, Australia; ^7^St. Vincent's Hospital, University of New South Wales, Sydney, NSW, Australia; ^8^Department of Haematology, Prince of Wales Hospital, University of New South Wales, Sydney, NSW, Australia; ^9^St. George Hospital, Kogarah, NSW, Australia; ^10^St. George Clinical School, University of New South Wales, Kogarah, NSW, Australia; ^11^Clinical Haematology, Monash Health and Monash University, Clayton, VIC, Australia; ^12^New South Wales Health Pathology, University of New South Wales, Sydney, NSW, Australia; ^13^Gosford Hospital, The University of Newcastle, Callaghan, NSW, Australia; ^14^Concord Repatriation General Hospital, University of Sydney, Concord, NSW, Australia; ^15^Liverpool Hospital, Western Sydney University, Liverpool, NSW, Australia; ^16^Border Medical Oncology Research Unit, Rural Medical School, Albury, NSW, Australia; ^17^School of Public Health and Preventive Medicine, Monash University, Melbourne, VIC, Australia; ^18^Precision Medicine, School of Clinical Sciences at Monash Health, Monash University, Melbourne, VIC, Australia; ^19^The Kirby Institute, University of New South Wales, Sydney, NSW, Australia

**Keywords:** follicular lymphoma, risk, survival, animal product, fish

## Abstract

**Background:**

The association between dietary intake of foods of animal origin and follicular lymphoma (FL) risk and survival is uncertain. In this study, we examined the relationship between dietary intake of dairy foods and fats, meat, fish and seafoods, and the likelihood of FL and survival.

**Methods:**

We conducted a population-based family case-control study in Australia between 2011 and 2016 and included 710 cases, 303 siblings and 186 spouse/partner controls. We assessed dietary intake of animal products prior to diagnosis (the year before last) using a structured food frequency questionnaire and followed-up cases over a median of 6.9 years using record linkage to national death data. We examined associations with the likelihood of FL using logistic regression and used Cox regression to assess association with all-cause and FL-specific mortality among cases.

**Results:**

We observed an increased likelihood of FL with increasing daily quantity of oily fish consumption in the year before last (highest category OR = 1.96, CI = 1.02–3.77; *p*-trend 0.06) among cases and sibling controls, but no associations with spouse/partner controls. We found no association between the likelihood of FL and the consumption of other types of fish or seafood, meats or dairy foods and fats. In FL cases, we found no association between meat or oily fish intake and all-cause or FL-specific mortality.

**Conclusion:**

Our study showed suggestive evidence of a positive association between oily fish intake and the likelihood of FL, but findings varied by control type. Further investigation of the potential role of environmental contaminants in oily fish on FL etiology is warranted.

## Introduction

Follicular lymphoma (FL) is an indolent type of non-Hodgkin lymphoma (NHL), accounting for 20–25% of incident NHLs diagnosed in Western countries (1). FL is most common in middle-aged and elderly individuals, with a median age at diagnosis of 61 years (2). The etiology of FL is not fully understood but risk increases with age, family history, smoking and pesticide exposure (3–5).

Food consumption represents a major route for exposure to chemical environmental contaminants, including pesticides. Findings from studies examining representative food samples have found high levels of organochlorine pesticides in foods of animal origin (6–10). The association between the consumption of animal-based products and FL risk has been examined in cohort (11–15) and case-control (16–20) studies with inconsistent findings. Most of the studies were limited by small sample size, variation in dietary assessment, changes in dietary habits and food composition over time, and recall bias. Whilst meta-analyses reported no association between FL risk and intake of red meat, white meat or processed meat, fish and seafoods, total dietary fat, poultry and eggs, or dairy products (21–27), some individual studies have observed dose-response trends and excess risk with the highest category of intake of poultry (13), red meat (18), “fat and meat” (14), and “meat, fat, and sweets” (17), whilst one study reported an inverse association with processed meat intake (13). The only prior study to examine the relationship between consumption of animal-based products and FL-specific mortality found an inverse association with the highest category of fish intake, but no association with the consumption of red meat, processed meat or dairy products (28).

To further investigate these associations, we carried out a population-based, family case-control study to examine the relationship between the dietary intake of dairy foods and fats, meat, fish, and seafoods, and the likelihood of FL and survival after FL diagnosis in an Australian cohort. We hypothesized the likelihood of FL may be associated with the consumption of animal products, and to a greater extent with fish than other food types given higher levels of organochlorine pesticide contamination in fish and seafoods compared to other foods of animal origin (6–8).

## Materials and methods

### Study sample

Eligible cases were aged between 20 and 74 years, resident in New South Wales (NSW) or Victoria, diagnosed with FL between 2011 and 2016, and ascertained following notification to the NSW or Victoria population-based cancer registry. Notification of new cancer cases to these registries are required by statute. Cases were eligible if they had histologically confirmed FL, no history of prior haematopoietic malignancy and provided informed consent. A total of 1,791 cases were identified. Of these, 213 cases with low confidence in the diagnosis based on pathology report review, underwent diagnostic slide review by an expert histopathologist (JT) (29), identifying 13 ineligible cases where the pathological diagnosis could not be confirmed. Of the remaining 1,778 eligible and contactable cases, 733 (41.2%) declined and 1,045 (58.8%) consented to be approached by the study coordinating center. Of those approached by the study, 77 cases could not be reached, 770 (79.5%) were enrolled and 198 (20.5%) declined. Of those enrolled, 710 cases (92.2%) completed the diet questionnaire (Supplementary Figure 1).

During recruitment, case participants were asked for consent to invite their family members to participate as controls in the study. Eligible controls were related (siblings) or unrelated (spouse/partner) family members of cases, aged between 20 and 74 years with no history of haematopoietic malignancy who were able to give informed consent. When a case had multiple siblings, those of the same sex and closest in the age were approached first. Where cases had no siblings or consenting siblings, they nominated their spouse/partner. Of those approached, 65 controls were unreachable for a response. A total of 517 (80.0%) controls were enrolled and 130 (20.0%) declined. The participation rate for sibling and spouse controls were 80.0 and 79.8%, respectively. Of those enrolled, 489 (94.6%) controls completed the diet questionnaire (Supplementary Figure 1).

Ethics approval for this study was obtained from the NSW Population and Health Services Research Ethics Committee (2011/07/337) and the Cancer Council Victoria Human Research Ethics Committee (HREC approval number 1114).

#### Dietary assessment

Participants completed a structured food frequency questionnaire adapted from a validated FFQ (30) focused on their usual dietary intake of animal products in the year before last (i.e., in the 2 years before enrolment for controls or FL diagnosis for cases) including: the type of margarine (none, butter blends, canola, olive oil, polyunsaturated, soy, sterol margarine), the type of milk [none, full milk, reduced fat milk (1–3% fat), skim milk (<1% fat)], the quantity of milk/day (none, <125 ml, about 125 ml, about 250 ml, about 500 ml, ≥750 ml), the frequency of intake of dairy foods and fats, meat, fish and seafood (none, <once/month, 1–3 times/month, 1 time/week, 2 times/week, 3–4 times/week, 5–6 times/week, 1 time a day, 2 times a day, ≥3 times a day). Types of dairy food included ricotta or cottage cheese, all other cheeses, cream or sour cream, ice cream and yogurt, while fat intake included oil, butter or margarine on cooked vegetables or salad dressing.

Participants recorded the type of meat consumed, including beef or veal, chicken, lamb, pork, sausages, processed meat and bacon, while fish and seafood intake included oily fish (fresh, smoked or tinned salmon, trout, herring, sardines, mackerel, eel), tuna, white fish (whiting, flathead, blue eye, ling, dory, flake), crustaceans (shrimp, prawns, crayfish, bugs, crabs), shellfish (oysters, mussels, scallops, clams, abalone), and other seafood (squid, cuttlefish, octopus). Participants reported the quantity (per serving) of fish and seafood usually consumed (none, <60 g, about 60 g, about 90 g, about 120 g, about 150 g, about 180 g, >180 g), with detailed information about the usual serving size for each type of fish and seafood. For meat, photographs of three different portion sizes were included in the questionnaire (Appendix 1). The portion sizes of meat were obtained from the Dietary Calibration Study, a sub sample of the Melbourne Collaborative Cohort Study (30).

We collected data on participants height, weight, and history of personal smoking at enrolment using a structured questionnaire. We calculated BMI (kg/m^2^) using the standard formula and categorized individuals as underweight (<18.5), normal weight (18.5–24.9), overweight (25.0–29.9), or obese (≥30) (31).

#### Case clinical and outcome data

We collected case clinical data from the treating clinicians including stage of disease (Ann Arbor criteria; I–IV), serum levels of lactate dehydrogenase ( ≤ or >institutional normal range), hemoglobin (<12 or ≥12 g/dL), number of areas of lymph node involvement (<5 or ≥5), β2-microglobulin ( ≤ or >normal range), largest nodal diameter ( ≤6 or >6 cm), and bone marrow involvement by lymphoma (no, yes, unknown) to allow the calculation of the Follicular Lymphoma International Prognostic Index (FLIPI/FLIPI-2) (32). Clinicians also provided the date and type of first-line treatment (none, radiotherapy, and/or chemotherapy). We extracted histologic grade (1-3B) from pathology reports.

We ascertained deaths to 05/11/2020 through probabilistic record linkage with the National Death Index by the Australian Institute of Health and Welfare.

### Statistical analysis

#### Likelihood of FL

We categorized the type of margarine as animal-based only, plant-based only, or animal- and plant-based. We classified the type of milk as full cream or low-fat milk. We then converted the volume (ml) of milk consumed into grams per day. We categorized the grams per day intake of all food types into tertiles using the distribution among exposed controls in each model. We used the never category (in the year before last) as the reference group in most analyses, while we used the highest category of meat intake as the reference group in the meat intake models.

We calculated the average grams of each type of meat consumed per day for each participant by multiplying the standard serving size (grams) (30) of each meat type (Appendix 2) by the scaled average portion size (Appendix 3) (30). We then multiplied the average weight of meat by the daily equivalent frequency (Appendix 4) to obtain the grams per day intake of each meat type. These values were summed to obtain the total daily quantity of meat consumed (grams per day). For fish and seafood, we calculated the grams per day intake of each type of fish or seafood by multiplying the quantity (grams) by the daily equivalent frequency (Appendix 4). We summed these values to obtain the total daily quantity of any fish or seafood intake (grams per day).

We estimated the odds ratios (ORs) and 95% confidence intervals (CI) for the association between dietary intake and FL risk among cases and matched sibling controls using conditional logistic regression models (33). We used the robust estimate of variance to allow for clustering within sibships. We also used unconditional logistic regression to estimate the association between dietary intake and FL risk using all cases and all spouse controls (33). We reviewed the literature and generated directed acyclic graphs (DAGs) (34) to guide the inclusion of confounders (Supplementary Figure 2). Our DAG suggested adjusting for smoking status (never, former, current), and we additionally adjusted for age (years), sex (male, female), ethnicity (Caucasian, others), and state (NSW, Victoria) in our multivariable models, based on our study design. We did not adjust for BMI because it is on the causal pathway between dietary intake and FL.

#### All-cause or FL-specific mortality

Follow-up of the cases began at the date of FL diagnosis and ended at death or end of follow-up (05/11/2020), whichever came first. We estimated hazard ratios (HRs) with 95% CI for all-cause and FL-specific mortality using Cox proportional hazard regression models. We used DAGs (Supplementary Figure 3) to guide inclusion of confounders, and adjusted for age, sex, ethnicity, and state in the basic models. We further adjusted for smoking status (never, former, current) in the fully adjusted model. We assessed the Cox proportional hazard assumption for all exposures and covariates using Schoenfeld residuals and observed no violation.

We performed multiple imputation (range 2–20) by chained equations under the assumption that missing values were missing at random (35). We performed two sensitivity analyses: restriction to cases and controls with no missing data in all models; cases matched to their spouse controls using conditional logistic regression. We tested the level of statistical significance for the categories of exposures (*p*-value). Where appropriate, we tested the linear trend of the associations with categorical variables by fitting the median value corresponding to each category and modeling this as a continuous variable. All statistical analyses were performed using STATA software, version 15.0 (STATA Corp., College Station, TX). All statistical tests analyses were two-sided and *P* < 0.05 was considered statistically significant.

## Results

Table 1 shows the characteristics of study participants. The median age was 60.8 [interquartile range (IQR) 52.5–67.1] years for cases, 59.3 (IQR 51.4–65.0) years for sibling controls, and 62.6 (53.9–68.3) years for spouse controls. A total of 468 cases had no sibling controls. Approximately 48% of cases and 59% of controls were female, and most (93%) were Caucasian. Data on types of margarine and cow milk, dairy foods and fats, meat and fish intake were missing for 1.7, 0.4, 4.4, 3.8 and 5.4% of participants, respectively.

**Table 1:** Characteristics of follicular lymphoma cases and controls.

**Characteristics**	**Cases *n* (%)**	**Controls**	
	**Sibling *n* (%)**	**Spouse *n* (%)**	
Total	710 (59.2)	303 (25.3)	186 (15.5)
**Sex**			
Male	369 (52.0)	123 (40.6)	76 (40.9)
Female	341 (48.0)	180 (59.4)	110 (59.1)
**Ethnicity**			
Caucasian/white	665 (93.7)	288 (95.1)	170 (91.4)
Other	19 (2.7)	8 (2.6)	6 (3.2)
Missing	26 (3.6)	7 (2.3)	10 (5.4)
**Smoking**			
Never	369 (52.0)	176 (58.1)	117 (62.9)
Current	66 (9.3)	21 (6.9)	13 (7.0)
Former	274 (38.6)	106 (35.0)	56 (30.1)
Missing	1 (0.1)	–	–
**Body mass index**			
< 18.5	3 (0.4)	2 (0.7)	–
18.5–24.9	201(28.3)	81 (26.7)	57 (30.7)
25.0–29.9	227 (32.0)	94 (31.0)	60 (31.7)
≥30	140 (19.7)	55 (18.2)	32 (17.2)
Missing	139 (19.6)	71 (23.4)	38 (20.4)
**Stage at diagnosis** ^a^			
I–II	181 (25.5)		
III–IV	349 (49.2)		
Missing	180 (25.3)		
**Histologic grade** ^a^			
1–2	488 (68.7)		
3A−3B^b^	150 (21.1)		
Missing	27 (3.8)		
Composite FL/DLBCL^c^	45 (6.3)		
**FLIPI score** ^a^			
Low (0–1)	179 (25.2)		
Intermediate (2)	123 (17.3)		
High (3,4)	140 (19.7)		
Missing	268 (37.8)		
**Treatment** ^a^			
None	166 (23.4)		
Chemotherapy	290 (40.8)		
Radiotherapy	46 (6.5)		
Chemotherapy/radiotherapy	31 (4.4)		
Missing	177 (24.9)		

a Cases only.

b Grade 3B = 45 cases.

c FL/DLBCL, follicular lymphoma and diffuse large B-cell lymphoma; FLIPI, follicular lymphoma international prognostic index.

### Likelihood of FL

#### Dairy foods and fats

In all models we observed no association between the likelihood of FL and the type of margarine, the daily quantity of cow milk intake, or the frequency of dairy foods and fats consumed in the year before last (Supplementary Tables 1, 2).

#### Meat consumption

We found no relationship between the likelihood of FL and the daily quantity of any type of meat consumed (Tables 2, 3).

**Table 2:** Odds ratios and 95% confidence intervals for the likelihood of FL in relation to meat consumed in the year before last among cases and sibling controls.

**Exposures**	**Cases^a^**	**Sibling controls^a^**	**OR (95% CI)^b^**	***P*-value**	***P*-trend**
**Daily quantity of meat intake (grams/day)** ^c^					
**Beef or veal (not corned)**					
>91.4	70	77	Ref.	0.61	0.52
91.4–3.10	69	98	0.71 (0.43–1.17)		
< 31.0	88	107	0.87 (0.51–1.47)		
Never	11	17	0.74 (0.27–2.02)		
**Chicken**					
>69.9	69	94	Ref.	0.87	0.55
69.9–32.6	90	101	1.14 (0.70–1.86)		
< 32.6	70	89	1.13 (0.67–1.88)		
Never	9	10	1.51 (0.46–4.95)		
**Lamb**					
>39.3	71	99	Ref.	0.64	0.34
39.3–18.4	61	74	1.19 (0.70–2.04)		
< 18.4	83	96	1.35 (0.78–2.35)		
Never	21	23	1.28 (0.58–2.84)		
**Pork (not corned or pickled)**					
>27.2	43	62	Ref.	0.23	0.77
27.2–12.3	61	76	1.42 (0.72–2.80)		
< 12.3	86	84	1.03 (0.72–2.04)		
Never	41	60	1.37 (0.64–2.93)		
**Sausages**					
>15.5	48	67	Ref.	0.57	0.24
15.5–7.8	69	92	1.45 (0.80–2.63)		
< 7.8	80	92	1.46 (0.76–2.79)		
Never	39	44	1.64 (0.77–3.47)		
**Processed meat (e.g., ham, corned beef, prosciutto, salami)**					
>17.1	48	75	Ref.	0.32	0.87
17.1–6.0	93	95	1.93 (0.81–3.39)		
< 6.0	77	97	1.54 (0.86–2.74)		
Never	17	32	0.83 (0.35–1.99)		
**Bacon**					
>6.9	63	84	Ref.	0.34	0.30
6.9–3.4	64	84	1.71 (0.94–3.10)		
< 3.4	81	96	1.48 (0.83–2.65)		
Never	31	35	1.45 (0.65–3.24)		
**Total daily quantity of any meat intake (grams/day)** ^c^ ^,^ ^d^					
>248.0	74	96	Ref.	0.92	0.60
248.0–120.0	82	98	1.19 (0.72–1.96)		
< 120.0	78	97	1.17 (0.67–2.03)		
Never	6	5	1.15 (0.31–4.29)		

a Cases and their matched related controls using conditional logistic regression models.

b Multivariable model: adjusted for age, sex, ethnicity, state and smoking status.

c Number of participants with missing data: daily quantity of meat intake (17), daily quantity of any meat intake (6).

d Total daily quantity of any meat intake was obtained by summing the daily grams per day of each type of meat.

**Table 3:** Odds ratios and 95% confidence intervals for the likelihood of FL in relation to meat consumed in the year before last among cases and spouse controls.

**Exposures**	**Cases^a^**	**Spouse controls^a^**	**OR (95% CI)^b^**	***P*-value**	***P*-trend**
**Daily quantity of meat intake (grams/day)** ^c^					
**Beef or veal (not corned)**					
>61.0	222	49	Ref.	0.18	0.59
61.0–30.5	187	64	1.63 (0.40–1.99)		
< 30.5	249	58	1.03 (0.66–1.61)		
Never	38	10	1.06 (0.47–2.37)		
**Chicken**					
>58.4	227	60	Ref.	0.64	0.31
58.4–32.7	224	58	1.12 (0.74–1.70)		
< 32.7	220	56	1.30 (0.84–2.02)		
Never	19	6	1.02 (0.37–2.79)		
**Lamb**					
>39.3	208	51	Ref.	0.59	0.28
39.3–18.4	181	56	0.84 (0.53–1.33)		
< 18.4	236	62	1.08 (0.71–1.64)		
Never	57	11	1.48 (0.71–3.06)		
**Pork (not corned or pickled)**					
>27.2	142	46	Ref.	0.17	0.15
27.2–12.3	187	50	1.40 (0.86–2.30)		
< 12.3	210	53	1.64 (0.90–2.68)		
Never	134	31	1.61 (0.91–2.67)		
**Sausages**					
>15.5	175	53	Ref.	0.74	0.44
15.5–7.8	200	48	1.58 (0.86–2.59)		
< 7.8	220	58	1.59 (0.81–2.51)		
Never	94	23	1.78 (0.71–3.17)		
**Processed meat (e.g., ham, corned beef, prosciutto, salami)**					
>17.1	173	47	Ref.	0.20	0.30
17.1–6.0	208	61	1.05 (0.65–1.71)		
< 6.0	238	61	1.28 (0.82–1.98)		
Never	69	12	0.50 (0.39–1.04)		
**Bacon**					
>6.9	198	56	Ref.	0.17	0.60
6.9–3.4	188	49	1.24 (0.77–1.98)		
< 3.4	227	63	1.35 (0.88–2.06)		
Never	84	12	0.70 (0.34–1.45)		
**Total daily quantity of any meat intake (grams/day)** ^c^ ^,^ ^d^					
>230.0	257	58	Ref.	0.16	0.55
230.0–127.6	194	64	0.74 (0.47–1.15)		
< 127.6	234	56	1.15 (0.74–1.79)		
Never	14	8	0.80 (0.26–2.43)		

a All cases and all spouse controls using unconditional logistic regression models.

b Multivariable model: adjusted for age, sex, ethnicity, state and smoking status.

c Number of participants with missing data: daily quantity of meat intake (34), daily quantity of any meat intake (12).

d Total daily quantity of any meat intake was obtained by summing the daily grams per day of each type of meat.

#### Fish and seafood consumption

We observed an elevated likelihood of FL with increasing daily quantity of oily fish consumed [highest category (>12.9 g/day) OR = 1.96, CI = 1.02–3.77 relative to never consumers; *p*-trend 0.06] among cases and sibling controls (Table 4), but no association in the analysis using spouse controls (Table 5). We found no association between the likelihood of FL and the intake of other fish or seafood types (Tables 4, 5).

**Table 4:** Odds ratios and 95% confidence intervals for the likelihood of FL in relation to fish and seafood consumed in the year before last among cases and sibling controls.

**Exposures**	**Cases^a^**	**Sibling controls^a^**	**OR (95% CI)^b^**	***P*-value**	***P*-trend**
**Daily quantity of fish and seafood intake (grams/day)** ^c^					
**Oily fish**					
Never	45	83	Ref.	0.02	0.06
< 8.6	80	72	2.14 (1.25–3.65)		
8.6–12.9	57	72	1.75 (0.93–3.30)		
>12.9	55	61	1.96 (1.02–3.77)		
**Tuna**					
Never	54	63	Ref.	0.35	0.28
< 6.0	59	85	0.88 (0.48–1.61)		
6.1–12.0	49	60	1.34 (0.70–2.54)		
>12.0	72	80	1.33 (0.70–2.52)		
**White fish**					
Never	14	27	Ref.	0.16	0.08
< 18.4	70	98	1.80 (0.74–4.33)		
18.4–39.3	81	97	1.81 (0.75–4.37)		
>39.3	72	71	2.55 (0.96–6.42)		
**Crustaceans**					
Never	68	85	Ref.	0.96	0.97
< 4.5	51	75	0.99 (0.54–1.82)		
4.5–9.0	59	69	1.04 (0.59–1.86)		
>9.0	47	50	0.95 (0.48–1.90)		
**Shellfish**					
Never	103	120	Ref.	0.11	0.26
< 3.0	59	56	1.39 (0.78–2.48)		
3.0–6.0	41	51	1.15 (0.62–2.14)		
>6.0	27	52	0.60 (0.30–1.20)		
**Other seafood**					
Never	84	110	Ref.	0.33	0.30
< 6.0	49	53	1.43 (0.79–2.57)		
6.0–17.1	39	49	1.26 (0.66–2.40)		
>17.1	39	40	1.40 (0.74–2.68)		
**Total daily quantity of fish or seafood intake (grams/day)** ^c^ ^,^ ^d^					
< 26.2	60	98	Ref.	0.46	0.18
26.2–51.5	95	96	1.24 (0.28–5.44)		
>51.5	82	96	2.46 (0.58–10.44)		
Never	4	11	2.44 (0.57–10.48)		

a Cases and their matched related controls using conditional logistic regression models.

b Multivariable model: adjusted for age, sex, ethnicity, state and smoking status.

c Number of participants with missing data: daily quantity of fish or seafood intake (41), total daily quantity of any fish or seafood intake (3).

d Total daily quantity of fish or seafood intake was obtained by summing the daily grams per day of each type of fish or seafood.

**Table 5:** Odds ratios and 95% confidence intervals for the likelihood of FL in relation to fish and seafood consumed in the year before last among cases and spouse controls.

**Exposures**	**Cases^a^**	**Spouse controls^a^**	**OR (95% CI)^b^**	***P*-value**	***P*-trend**
**Daily quantity of fish and seafood intake (grams/day)** ^c^					
**Oily fish**					
Never	132	33	Ref.	0.89	0.49
< 7.5	193	51	0.96 (0.59–1.57)		
7.5–12.9	192	51	0.91 (0.57–1.47)		
>12.9	166	47	0.86 (0.52–1.40)		
**Tuna**					
Never	157	34	Ref.	0.43	0.25
< 8.6	193	51	0.72 (0.46–1.15)		
8.6–12.0	116	46	0.49 (0.30–1.18)		
>12.0	211	48	0.89 (0.75–1.43)		
**White fish**					
Never	42	12	Ref.	0.23	0.89
< 9.0	200	58	1.08 (0.49–2.37)		
9.0–15.0	228	62	1.00 (0.46–2.13)		
>15.0	214	52	1.09 (0.51–2.38)		
**Crustaceans**					
Never	180	43	Ref.	0.81	0.45
< 4.5	138	47	0.67 (0.40–1.12)		
4.5–9.0	185	46	0.77 (0.46–1.27)		
>9.0	154	38	0.83 (0.52–1.34)		
**Shellfish**					
Never	286	75	Ref.	0.49	0.27
< 3.0	144	40	0.86 (0.56–1.31)		
3.0–6.0	123	31	0.83 (0.52–1.33)		
>6.0	109	32	0.79 (0.49–1.27)		
**Other seafood**					
Never	273	75	Ref.	0.51	0.43
< 4.5	119	33	0.94 (0.60–1.44)		
4.5–6.0	100	31	0.72 (0.43–1.18)		
>6.0	129	31	0.93 (0.58–1.49)		
**Total daily quantity of fish or seafood intake (grams/day)** ^c^ ^,^ ^d^					
< 32.2	235	60	Ref.	0.92	0.98
32.2–53.2	216	60	1.15 (0.37–3.62)		
>53.2	238	59	1.02 (0.32–3.20)		
Never	18	7	1.14 (0.36–3.57)		

a All cases and all spouse controls using unconditional logistic regression models.

b Multivariable model: adjusted for age, sex, ethnicity, state and smoking status.

c Number of participants with missing data: daily quantity of fish or seafood intake (83), total daily quantity of any fish or seafood intake (3).

d Total daily quantity of fish or seafood intake was obtained by summing the daily grams per day of each type of fish or seafood.

### Case all-cause and FL-specific mortality

The median follow-up period was 6.9 (IQR = 5.8–8.2) years. During follow-up, a total of 48 (6.8%) cases died, and of these 22 (45.8%) were FL-related deaths. We found no association between the meat or oily fish intake and all-cause or FL-specific mortality (Table 6).

**Table 6:** Hazard ratios and 95% confidence intervals for all-cause and FL-specific mortality in relation to dietary pattern 2 years prior to enrolment.

**Exposure**	**Person-months**	**All-cause mortality**			**FL-specific mortality**		
		**No. of deaths**	**HR (95% CI)** ^a^	***P*** **-value**	**No. of deaths**	**HR (95% CI)** ^a^	***P*** **-value**
**Meat intake (grams/day)**							
>185.2	27,384	26	Ref.	0.84	12	Ref.	0.75
≤ 185.2	29,730	22	0.94 (0.51–1.72)		10	0.86 (0.34–2.16)	
None	–	–	–		–	–	
**Oily fish intake (grams/day)**							
>9.0	22,480	14	Ref.	0.32	6	Ref.	0.50
≤ 9.0	23,272	24	1.64 (0.84–3.20)		11	1.63 (0.58–4.51)	
None	11,016	10	1.60 (0.70–3.63)		5	1.06 (0.59–6.60)	
			*P*-trend 0.20			*P*-trend 0.16	

^a^Multivariable model—adjusted for age, sex, ethnicity, state and smoking status.

### Sensitivity analyses

Results from imputed analyses were consistent with findings without imputations (Supplementary Tables 3a–f). Findings from matched case-spouse control pairs using conditional regression showed similar ORs with the unconditional logistic regression models with all cases and all spouse controls (Supplementary Tables 4a–c).

## Discussion

In this population-based family case-control study, we observed an elevated likelihood of FL with increasing daily quantity of oily fish intake for cases compared to matched sibling controls. We found no association between the likelihood of FL and the consumption of other types of fish or seafood, meats or dairy foods and fats. Overall, the odds ratios for cases and spouse controls appeared attenuated toward the null for most exposures compared to the case-sibling pairs. We found no association between meat intake and case all-cause or FL-specific mortality.

Our findings of a positive association with the intake of oily fish and likelihood of FL contrasts with results from the only similar prior study (19). Chang et al. (19) in a Swedish case-control study, observed no association between FL risk and the highest category of fatty fish intake (≥3 servings/day of salmon, mackerel, herring; *n* = 4 exposed cases) or any fish intake (≥3 servings/day; *n* = 11 exposed cases) 2 years before enrolment. The null finding with oily fish intake (and other types of animal-based food products) among cases and spouse controls in our study may be due to the strong concordance of dietary patterns between spouses compared to between adult siblings (36). Spouses tend to influence each other's diet and eating behavior (37–39). Results from a cohort study examining the association between FL risk and any fish intake was also null (11). Meanwhile, Daniel et al. (12) in the US National Institutes of Health and the American Association of Retired Persons (NIH-AARP) Diet and Health Study observed that any fish intake above the first quintile (Q1) appeared to be associated with an increased risk of FL (e.g., HR = 1.41, CI = 1.10–1.82; Q2 vs. Q1) but risk was attenuated across the upper categories of intake (HR = 1.27, CI = 0.97–1.65; *p*-trend = 0.19; Q5 vs. Q1).

Given that fish is considered part of a healthy diet, definitive evidence would be required to recommend reduction of consumption. Fish oil contains omega−3 polyunsaturated fatty acids which have been shown to have a beneficial effect in reducing inflammatory and cardiovascular diseases (40, 41). However, fat-soluble environmental contaminants may concentrate in fatty tissues of fish through bioaccumulation and biomagnification processes (42). Organochlorine pesticides (OCPs) were produced and used in Australia until the early 1990s when their use was largely phased out (43). Even though OCPs have been banned for 30 years, their lipophilic properties and long half-lives allows them to bioaccumulate and persist in the environment, providing opportunities for continued exposure through dietary, environmental and occupational sources (44). Several studies have documented a higher level of OCP contamination in fish and seafoods compared to other foods of animal origin (6–8). Results from a Swedish study that measured OCP concentrations in six food groups (fish, meat, dairy products, egg, fats/oils, and pastries) showed higher levels of dichlorodiphenyldichloroethylene (DDE) and dichlorodiphenyltrichloroethane (DDT) in fish compared to the other food groups (7), similar to findings from Australian (6) and US (8) studies that examined OCPs in nine food groups and 31 food types, respectively. Furthermore, findings from occupational studies showed an elevated FL risk with organochlorine pesticide exposure (45–47), while meta-analyses of observational studies showed a positive association between plasma organochlorine DDE (5) and self-reported DDT (48) exposure, and FL risk.

We found no association between the likelihood of FL and intake of dairy foods and fats or meat, consistent with some cohort (12, 15) and case-control (16, 19, 20) studies. In contrast, Rohrmann et al. (13) in the European Prospective Investigation into Cancer and Nutrition Study found an increased risk of FL with the highest category of poultry intake (*n* = 29; HR = 1.80, CI = 1.07–3.04, ≥40 g/day vs. <10 g/day), and an inverse association with the highest category of processed meat consumption (HR = 0.31, CI = 0.10–0.94, ≥80 g/day vs. <20 g/day), based on four exposed cases; the trends in risk for increasing poultry and processed meat intake were statistically significant. Erber et al. (14) in the US multiethnic cohort study reported a significant trend and elevated FL risk the highest category of “fat and meat” intake (HR = 5.16, CI = 1.33–20.00) in men based on a small number of exposed cases (*n* = 12) but no association for women. Similarly, two small US case-control studies (17, 18) reported a dose-response and increased risk with high intakes of meat and fat.

We found no association between meat or oily fish intake and all-cause or FL-specific mortality, partly consistent with the only prior study (28). Leo et al. (28) in the US multiethnic cohort study reported no association between FL-specific mortality and red meat, processed meat or dairy product intake, and an inverse association with the highest category of fish intake (HR = 0.29, CI = 0.13–0.64; ≥9.2 g/4,184 KJ/day).

To our knowledge, no previous studies have used a population-based family design to investigate the relationship between dietary exposures and likelihood of FL. Family members are usually more willing to participate as controls compared with traditional case-control studies, thus reducing potential bias that may arise from non-participation (49). All cases were histologically confirmed and linked to the NSW and Victoria population-based cancer registries for affirmation. In addition, we used a validated food frequency questionnaire (FFQ) for the assessment of diet (30).

Our study has several limitations. There is possible correlation of exposure between cases and spouse/partner controls as they are more likely to live together and share similar diets, causing the odds ratios for these case/control pairs to be biased toward the null (37, 39). There is also possible correlation of exposures among cases and sibling controls as they likely grew up in the same childhood environment, thus may share similar dietary or eating habits (50). Whilst we accounted for clustering within sibships in our analyses, the concordance as expected was stronger among cases and spouse controls because we assessed recent diet and not total lifetime exposure. We acknowledge that not all those who were eligible agreed to participate and the non-participation may have biased our results. As typical of studies using FFQ to assess dietary intake, reliance on the participants' memory to recall history of food consumed may have resulted in measurement error and biased our odds ratios toward the null. This is particularly the case for fish and seafood where text descriptions of serving sizes were given, while pictures were provided for meat serving sizes. In addition, it is possible cooking procedures may reduce contaminants in foods (51), and we did not collect data on cooking methods. We also acknowledge the multiplicity of our analyses may have resulted in chance findings, despite our *a priori* focus on animal products rather than a full dietary assessment. Lastly, we could not assess associations with FL-specific mortality due to the small number of cases.

In conclusion, our findings are consistent with most previous observational studies in showing no association with the intake of meat, dairy foods and fats, and likelihood of FL and survival. Our study showed suggestive evidence of an association between oily fish intake and increased likelihood of FL. Further prospective studies or pooled analyses are needed to comprehensively evaluate the role of environmental contaminants in oily fish on the etiology FL.

## Data availability statement

The raw data supporting the conclusions of this article will be made available by the authors, without undue reservation.

## Ethics statement

The studies involving human participants were reviewed and approved by NSW Population and Health Services Research Ethics Committee (2011/07/337) and Cancer Council Victoria Human Research Ethics Committee (HREC approval number 1114). The patients/participants provided their written informed consent to participate in this study.

## Author contributions

ML, JTu, FB, JS, HP, SM, MHe, JTr, SO, RL, FR, EV, MHa, CT, CU, GB, GG, and CV: conceptualization. ML, MO, JB, JTu, FB, JS, HP, SM, MHe, JTr, SO, RL, FR, EV, MHa, CT, CU, GB, GG, and CV: methodology and writing—review and editing. MO: formal analysis and writing—original draft preparation. ML and CV: supervision. CV: project administration and funding acquisition. All authors have read and agreed to the published version of the manuscript.
