# Supplementary material for: Dietary intake of animal-based products and likelihood of follicular lymphoma and survival: A population-based family case-control study
Source: Front Nutr. 2023 Jan 4;9:1048301. doi: 10.3389/fnut.2022.1048301 (PMC9846614; doi:10.3389/fnut.2022.1048301)
Supplement: Supplementary file 1 [file Data_Sheet_1.docx]

**SUPPLEMENTARY FILE**

| Eligible study population  Cases (n = 1778)  Controls (n = 652)  Declined to be approached by study  (n = 733 cases)  Excluded    Consented to being approached by study Cases (n = 1045)  Controls (n = 647)  Not reachable (n = 77 cases)  Declined to participate  (n = 198 cases, 130 controls)    Excluded    Completed smoking and alcohol questionnaire  Cases (n = 710)  Controls (n = 489)  Did not complete early-life and body size questionnaire  (n = 60 cases, 28 controls)  Not reachable (n = 77)  Excluded  Enrolled Cases (n = 770)  Controls (n = 517) |
| --- |

Supplementary figure 1: Flowchart of recruitment to the diet study


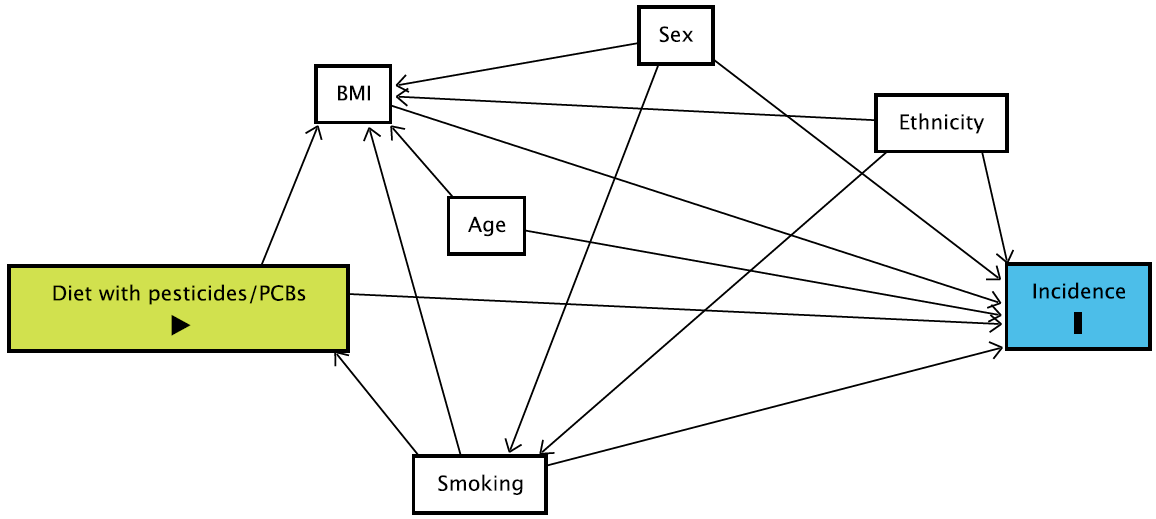


Supplementary figure 2: Potential confounders of the association between diet and likelihood of FL

Note: We hypothesized that dietary intake of food contaminated with pesticides or polychlorinated biphenyls (PCBs) may increase the likelihood of FL.

Evidence underpinning associations included in the directed acyclic graph:

Pesticides/PCBs and diet – Levels of pesticides dichlorodiphenyldichloroethylene (DDT) and PCBs in composite dairy and fish products (1)

BMI and FL – Modest positive association with obesity as an adult (2)

BMI and diet – Overweight and obesity is associated with western/unhealthy dietary pattern (3)

Smoking status and BMI – Smoking frequency is positively associated with BMI (4). BMI also increases the risk of being a smoker (5)

Smoking status and diet – Smoking is associated with poor diet quality and lower BMI (6, 7)

BMI and age – Higher prevalence of obesity in 65-75 years age group in Australia compared to other age groups (8)

BMI and sex – Higher prevalence rate of overweight and obesity in men than women in Australia (8)

BMI and ethnicity – Higher prevalence of obesity in Blacks compared to Caucasians or other groups (9)


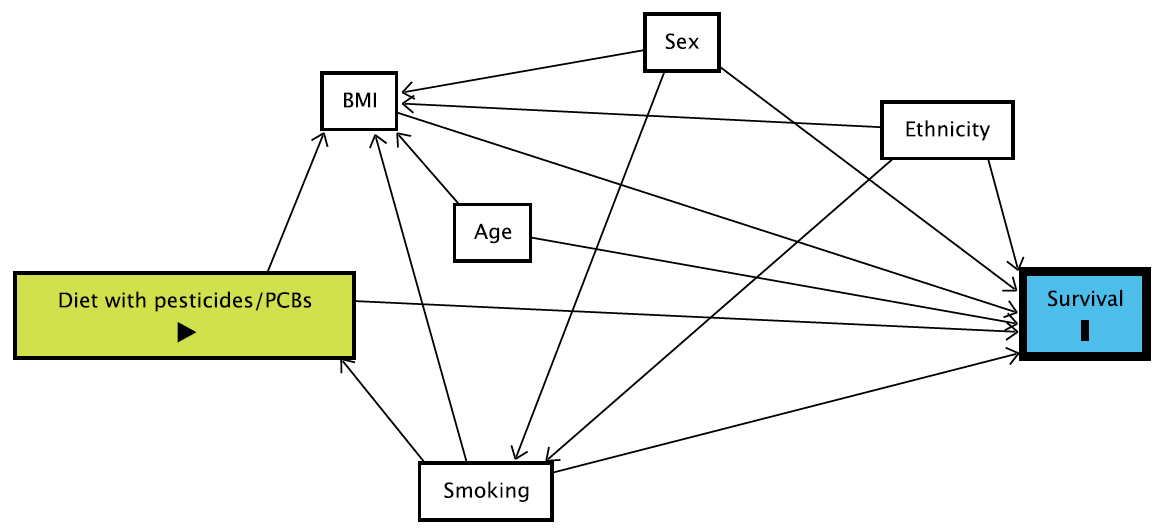
 Supplementary figure 3: Potential confounders of the association between diet and FL survival

Note: We hypothesized that dietary intake of food contaminated with pesticides/PCBs may increase FL mortality.

Evidence underpinning associations included in directed acyclic graph:

Age and FL survival – Better FL survival among males than females (10)

Sex and FL survival – Better FL survival among age group <60 years compared to other age groups (10)

Ethnicity and FL survival – Better FL survival among Caucasians compared to other groups (10)

Smoking and FL survival – Poorer FL survival with smoking (11)

BMI and FL survival – Poorer FL survival with lower BMI (12)

BMI and diet – Overweight and obesity is associated with western/unhealthy dietary pattern (3)

BMI and age – Higher prevalence of obesity in 65-75 years age group in Australia compared to other age groups (8)

BMI and sex – Higher prevalence rate of overweight and obesity in men than women in Australia (8)

BMI and ethnicity – Higher prevalence of obesity in Blacks compared to Caucasians or other groups (9)

Smoking status and age – Higher smoking prevalence in 40-49 years age group in Australia compared to other age groups (13)

Smoking status and sex – Higher smoking prevalence in men than women (14)

Smoking status and ethnicity – Higher prevalence among Whites compared to Black populations (15)

Smoking status and BMI – Smoking frequency is positively associated with BMI (29). BMI also increases the risk of being a smoker (5)

Smoking status and diet – Smoking is associated with poor diet quality and lower BMI (6, 7)

**Supplementary Table 1: Odds ratios and 95% confidence intervals for the likelihood of FL in relation to dairy foods and fats consumed in the year before last among cases and sibling controls**

| Exposures | Cases ^a^ | Sibling controls ^a^ | OR (95% CI) ^b^ | P value | P trend |
| --- | --- | --- | --- | --- | --- |
| Margarine type ^c^ |  |  |  |  |  |
| Never | 46 | 70 | Ref. | 0.36 | 0.10 |
| Animal-based only | 37 | 55 | 0.98 (0.47-2.23) |  |  |
| Plant-based only | 116 | 141 | 1.30 (0.74-2.29) |  |  |
| Animal- and plant-based | 41 | 34 | 1.73 (0.85-3.50) |  |  |
| Daily quantity of full cream cow milk intake (grams/day) | | | | |  |
| ≤128.7 | 43 | 58 | Ref. | 0.78 | 0.56 |
| 128.7-257.5 | 29 | 45 | 0.56 (0.11-2.96) |  |  |
| >257.5 | 26 | 35 | 0.92 (0.31-2.71) |  |  |
| Daily quantity of low fat (≤3% fat) cow milk intake (grams/day) | | | | |  |
| ≤128.7 | 52 | 94 | Ref. | 0.13 | 0.22 |
| 128.7-257.5 | 62 | 50 | 1.42 (0.60-3.44) |  |  |
| >257.5 | 45 | 43 | 2.82 (0.92-6.18) |  |  |
| Frequency of dairy and fat consumption ^c^ | | | | |  |
| Margarine on cooked vegetables | | | | |  |
| Never | 144 | 179 | Ref. | 0.56 | 0.42 |
| <1 time/month | 30 | 34 | 0.79 (0.40-1.51) |  |  |
| 1-3 times/month – 2 times/week | 34 | 43 | 0.79 (0.41-1.52) |  |  |
| >2 times/week | 25 | 35 | 1.34 (0.71-2.55) |  |  |
| Butter/dairy blend on cooked vegetables | | | | |  |
| Never | 129 | 157 | Ref. | 0.89 | 0.76 |
| <1 time/month | 34 | 46 | 0.96 (0.52-1.74) |  |  |
| 1-3 times/month – 1 time/week | 36 | 45 | 0.95 (0.54-1.66) |  |  |
| >1 time/week | 35 | 40 | 1.21 (0.62-2.36) |  |  |
| Mayonnaise |  |  |  |  |  |
| Never | 59 | 71 | Ref. | 0.80 | 0.70 |
| <1 time/month | 80 | 93 | 1.24 (0.72-2.11) |  |  |
| 1-3 times/month | 45 | 62 | 0.95 (0.50-1.83) |  |  |
| >1-3 time/month | 56 | 68 | 1.21 (0.66-2.24) |  |  |
| Ricotta or cottage cheese |  |  |  |  |  |
| Never | 101 | 133 | Ref. | 0.59 | 0.47 |
| <1 time/month | 64 | 86 | 1.08 (0.53-2.20) |  |  |
| 1-3 times/month | 42 | 37 | 1.18 (0.71-1.98) |  |  |
| >1-3 time/month | 31 | 34 | 1.57 (0.82-3.01) |  |  |
| All other cheeses |  |  |  |  |  |
| Never | 5 | 9 | Ref. | 0.52 | 0.75 |
| ≤2 times/week | 80 | 105 | 1.37 (0.43-4.39) |  |  |
| 2 – 3-4 times/week | 110 | 122 | 1.74 (0.55-5.55) |  |  |
| >3-4 times/week | 42 | 59 | 1.24 (0.36-4.33) |  |  |
| Cream or sour cream |  |  |  |  |  |
| Never | 40 | 54 | Ref. | 0.47 | 0.16 |
| <1 time/month | 62 | 88 | 1.38 (0.75-2.53) |  |  |
| 1-3 times/month | 73 | 88 | 1.45 (0.79-2.67) |  |  |
| >1-3 time/month | 65 | 65 | 1.64 (0.86-3.13) |  |  |
| Ice-cream |  |  |  |  |  |
| Never | 17 | 29 | Ref. | 0.15 | 0.10 |
| <1 time/week | 13 | 151 | 2.59 (0.84-5.99) |  |  |
| 1 time/week | 35 | 42 | 2.29 (0.84-5.58) |  |  |
| >1 time/week | 58 | 74 | 2.46 (0.86-6.30) |  |  |
| Yogurt |  |  |  |  |  |
| Never | 29 | 39 | Ref. | 0.53 | 0.21 |
| <3-4 times/week | 66 | 92 | 1.18 (0.61-2.89) |  |  |
| 3-4 – 5-6 times/week | 89 | 95 | 1.47 (0.72-3.00) |  |  |
| >5-6 times/week | 56 | 69 | 1.51 (0.78-2.92) |  |  |

^a^ Cases and their matched related controls using conditional logistic regression models

^b^ Multivariable model: adjusted for age, sex, ethnicity, state and smoking status

^c^ Number of participants with missing data: margarine type consumed (5), frequency of dairy and fat consumption (23)

**Supplementary Table 2: Odds ratios and 95% confidence intervals for the likelihood of FL in relation to dairy foods and fats consumed in the year before last among cases and spouse controls**

| Exposures | Cases ^a^ | Spouse controls ^a^ | OR (95% CI) ^b^ | P value | P trend |
| --- | --- | --- | --- | --- | --- |
| Margarine type ^c^ | | | | |  |
| Never | 131 | 39 | Ref. | 0.26 | 0.23 |
| Animal-based only | 116 | 27 | 1.43 (0.82-2.49) |  |  |
| Plant-based only | 337 | 90 | 1.19 (0.78-1.82) |  |  |
| Animal- and plant-based | 113 | 26 | 1.56 (0.89-2.75) |  |  |
| Daily quantity of full cream cow milk intake (grams/day) | | | | |  |
| ≤128.7 | 128 | 37 | Ref. | 0.86 | 0.84 |
| 128.7-257.5 | 83 | 22 | 0.75 (0.14-4.21) |  |  |
| >257.5 | 101 | 18 | 1.22 (0.18-8.09) |  |  |
| Daily quantity of low fat (≤3% fat) cow milk intake (grams/day) | | | | |  |
| ≤128.7 | 143 | 45 | Ref. | 0.33 | 0.52 |
| 128.7-257.5 | 168 | 45 | 1.40 (0.58-3.36) |  |  |
| >257.5 | 130 | 34 | 1.97 (0.80-4.84) |  |  |
| Frequency of dairy and fat consumption ^c^ | | | | |  |
| Margarine on cooked vegetables | | | | |  |
| Never | 402 | 106 | Ref. | 0.96 | 0.75 |
| <1 time/month | 74 | 24 | 0.94 (0.56-1.59) |  |  |
| 1-3 times/month – 1 time/week | 97 | 22 | 0.99 (0.59-1.68) |  |  |
| >1 time/week | 106 | 28 | 0.91 (0.57-1.47) |  |  |
| Butter/dairy blend on cooked vegetables | | | | |  |
| Never | 382 | 97 | Ref. | 0.66 | 0.84 |
| <1 time/month | 82 | 26 | 0.88 (0.53-1.47) |  |  |
| 1-3 times/month – 1 time/week | 102 | 23 | 1.16 (0.67-1.99) |  |  |
| >1 time/week | 114 | 32 | 0.90 (0.58-1.39) |  |  |
| Mayonnaise |  |  |  |  |  |
| Never | 168 | 39 | Ref. | 0.78 | 0.29 |
| <1 time/month | 188 | 46 | 0.91 (0.57-1.47) |  |  |
| 1-3 times/month | 159 | 40 | 0.88 (0.53-1.46) |  |  |
| >1-3 time/month | 177 | 55 | 0.78 (0.49-1.24) |  |  |
| Ricotta or cottage cheese |  |  |  |  |  |
| Never | 315 | 84 | Ref. | 0.49 | 0.77 |
| <1-3 times/month | 175 | 42 | 1.19 (0.78-1.81) |  |  |
| 1-3 times/month | 110 | 36 | 0.87 (0.55-1.38) |  |  |
| >1-3 time/month | 78 | 19 | 0.99 (0.57-1.73) |  |  |
| All other cheeses |  |  |  |  |  |
| Never | 25 | 3 | Ref. | 0.40 | 0.33 |
| <3-4 times/week | 363 | 98 | 0.53 (0.16-1.74) |  |  |
| 3-4 times/week | 181 | 50 | 0.49 (0.15-1.63) |  |  |
| >3-4 times/week | 120 | 33 | 0.48 (0.14-1.60) |  |  |
| Cream or sour cream |  |  |  |  |  |
| Never | 134 | 25 | Ref. | 0.52 | 0.24 |
| <1-3 times/month | 166 | 45 | 0.74 (0.43-1.25) |  |  |
| 1-3 times/month | 196 | 54 | 0.69 (0.42-1.16) |  |  |
| >1-3 time/month | 197 | 56 | 0.73 (0.44-1.21) |  |  |
| Ice-cream |  |  |  |  |  |
| Never | 52 | 14 | Ref. | 0.24 | 0.68 |
| <1-3 times/month | 162 | 40 | 0.84 (0.44-1.61) |  |  |
| 1-3 times/month – 1 time/week | 288 | 89 | 1.11 (0.54-2.27) |  |  |
| >1 time/week | 194 | 40 | 1.25 (0.62-2.50) |  |  |
| Yogurt |  |  |  |  |  |
| Never | 112 | 33 | Ref. | 0.10 | 0.31 |
| <2 times/week | 210 | 57 | 1.22 (0.75-2.02) |  |  |
| 2 – 3-4 times/week | 234 | 46 | 1.77 (0.93-3.03) |  |  |
| >3-4 times/week | 139 | 43 | 1.20 (0.71-2.02) |  |  |

^a^ All cases and all spouse controls using unconditional logistic regression models

^b^ Multivariable model: adjusted for age, sex, ethnicity, state and smoking status

^c^ Number of participants with missing data: margarine type consumed (17), frequency of dairy and fat consumption (38)

**Supplementary Table 3a: Odds ratios and 95% confidence intervals for the likelihood of FL in relation to dairy foods and fats consumed in the year before last among cases and sibling controls (no imputation)**

| Exposures | Cases ^a^ | Sibling controls ^a^ | OR (95% CI) ^b^ | P value | P trend |
| --- | --- | --- | --- | --- | --- |
| Margarine type |  |  |  |  |  |
| Never | 46 | 70 | Ref. | 0.36 | 0.09 |
| Animal-based only | 37 | 55 | 0.99 (0.49-2.01) |  |  |
| Plant-based only | 116 | 141 | 1.30 (0.74-2.29) |  |  |
| Animal- and plant-based | 41 | 34 | 1.76 (0.87-3.56) |  |  |
| Daily quantity of full cream cow milk intake (grams/day) | | | | |  |
| ≤128.7 | 43 | 58 | Ref. | 0.78 | 0.56 |
| 128.7-257.5 | 29 | 45 | 0.56 (0.11-2.96) |  |  |
| >257.5 | 26 | 35 | 0.92 (0.31-2.71) |  |  |
| Daily quantity of low fat (≤3% fat) cow milk intake (grams/day) | | | | |  |
| ≤128.7 | 52 | 94 | Ref. | 0.13 | 0.22 |
| 128.7-257.5 | 62 | 50 | 1.42 (0.60-3.44) |  |  |
| >257.5 | 45 | 43 | 2.82 (0.92-6.18) |  |  |
| Frequency of dairy and fat consumption | | | | |  |
| Margarine on cooked vegetables | | | | |  |
| Never | 144 | 179 | Ref. | 0.33 | 0.39 |
| <1 time/month | 30 | 34 | 0.74 (0.38-1.45) |  |  |
| 1-3 times/month – 2 times/week | 34 | 43 | 0.77 (0.40-1.48) |  |  |
| >2 times/week | 25 | 35 | 1.41 (0.72-2.74) |  |  |
| Butter/dairy blend on cooked vegetables | | | | |  |
| Never | 129 | 157 | Ref. | 0.82 | 0.52 |
| <1 time/month | 34 | 46 | 0.92 (0.51-1.67) |  |  |
| 1-3 times/month – 1 time/week | 36 | 45 | 1.02 (0.57-1.82) |  |  |
| >1 time/week | 35 | 40 | 1.34 (0.68-2.66) |  |  |
| Mayonnaise |  |  |  |  |  |
| Never | 59 | 71 | Ref. | 0.59 | 0.83 |
| <1 time/month | 80 | 93 | 1.25 (0.77-2.34) |  |  |
| 1-3 times/month | 45 | 62 | 0.92 (0.47-1.79) |  |  |
| >1-3 time/month | 56 | 68 | 1.19 (0.64-2.22) |  |  |
| Ricotta or cottage cheese |  |  |  |  |  |
| Never | 101 | 133 | Ref. | 0.48 | 0.33 |
| <1 time/month | 64 | 86 | 1.18 (0.57-2.43) |  |  |
| 1-3 times/month | 42 | 37 | 1.16 (0.85-1.99) |  |  |
| >1-3 time/month | 31 | 34 | 1.31 (0.77-2.22) |  |  |
| All other cheeses |  |  |  |  |  |
| Never | 5 | 9 | Ref. | 0.33 | 0.52 |
| ≤2 times/week | 80 | 105 | 1.39 (0.61-4.32) |  |  |
| 2 – 3-4 times/week | 110 | 122 | 1.98 (0.78-5.46) |  |  |
| >3-4 times/week | 42 | 59 | 1.23 (0.53-4.35) |  |  |
| Cream or sour cream |  |  |  |  |  |
| Never | 40 | 54 | Ref. | 0.58 | 0.20 |
| <1 time/month | 62 | 88 | 1.35 (0.73-2.50) |  |  |
| 1-3 times/month | 73 | 88 | 1.43 (0.77-2.64) |  |  |
| >1-3 time/month | 65 | 65 | 1.58 (0.82-3.06) |  |  |
| Ice-cream |  |  |  |  |  |
| Never | 17 | 29 | Ref. | 0.18 | 0.17 |
| <1 time/week | 13 | 151 | 2.43 (0.82-5.68) |  |  |
| 1 time/week | 35 | 42 | 1.86 (0.76-4.53) |  |  |
| >1 time/week | 58 | 74 | 2.17 (0.88-6.47) |  |  |
| Yogurt |  |  |  |  |  |
| Never | 29 | 39 | Ref. | 0.67 | 0.24 |
| <3-4 times/week | 66 | 92 | 1.15 (0.59-2.25) |  |  |
| 3-4 – 5-6 times/week | 89 | 95 | 1.42 (0.73-2.77) |  |  |
| >5-6 times/week | 56 | 69 | 1.45 (0.70-2.99) |  |  |

^a^ Cases and their matched related controls using conditional logistic regression models

^b^ Multivariable model: adjusted for age, sex, ethnicity, state and smoking status

**Supplementary Table 3b: Odds ratios and 95% confidence intervals for the likelihood of FL in relation to dairy foods and fats consumed in the year before last among cases and spouse controls (no imputation)**

| Exposures | Cases ^a^ | Spouse controls ^a^ | OR (95% CI) ^b^ | P value | P trend |
| --- | --- | --- | --- | --- | --- |
| Margarine type | | | | |  |
| Never | 131 | 39 | Ref. | 0.36 | 0.26 |
| Animal-based only | 116 | 27 | 1.47 (0.85-2.53) |  |  |
| Plant-based only | 337 | 90 | 1.18 (0.77-1.79) |  |  |
| Animal- and plant-based | 113 | 26 | 1.54 (0.88-2.71) |  |  |
| Daily quantity of full cream cow milk intake (grams/day) | | | | |  |
| ≤128.7 | 128 | 37 | Ref. | 0.86 | 0.84 |
| 128.7-257.5 | 83 | 22 | 0.75 (0.14-4.21) |  |  |
| >257.5 | 101 | 18 | 1.22 (0.18-8.09) |  |  |
| Daily quantity of low fat (≤3% fat) cow milk intake (grams/day) | | | | |  |
| ≤128.7 | 143 | 45 | Ref. | 0.33 | 0.52 |
| 128.7-257.5 | 168 | 45 | 1.40 (0.58-3.36) |  |  |
| >257.5 | 130 | 34 | 1.97 (0.80-4.84) |  |  |
| Frequency of dairy and fat consumption | | | | |  |
| Margarine on cooked vegetables | | | | |  |
| Never | 402 | 106 | Ref. | 0.97 | 0.77 |
| <1 time/month | 74 | 24 | 0.91 (0.53-1.57) |  |  |
| 1-3 times/month – 1 time/week | 97 | 22 | 1.00 (0.59-1.70) |  |  |
| >1 time/week | 106 | 28 | 0.92 (0.57-1.49) |  |  |
| Butter/dairy blend on cooked vegetables | | | | |  |
| Never | 382 | 97 | Ref. | 0.85 | 0.90 |
| <1 time/month | 82 | 26 | 0.88 (0.52-1.47) |  |  |
| 1-3 times/month – 1 time/week | 102 | 23 | 1.16 (0.68-1.99) |  |  |
| >1 time/week | 114 | 32 | 0.91 (0.59-1.41) |  |  |
| Mayonnaise |  |  |  |  |  |
| Never | 168 | 39 | Ref. | 0.71 | 0.25 |
| <1 time/month | 188 | 46 | 0.95 (0.58-1.53) |  |  |
| 1-3 times/month | 159 | 40 | 0.88 (0.53-1.45) |  |  |
| >1-3 time/month | 177 | 55 | 0.78 (0.49-1.24) |  |  |
| Ricotta or cottage cheese |  |  |  |  |  |
| Never | 315 | 84 | Ref. | 0.75 | 0.87 |
| <1-3 times/month | 175 | 42 | 1.19 (0.78-1.81) |  |  |
| 1-3 times/month | 110 | 36 | 0.89 (0.56-1.41) |  |  |
| >1-3 time/month | 78 | 19 | 1.01 (0.58-1.78) |  |  |
| All other cheeses |  |  |  |  |  |
| Never | 25 | 3 | Ref. | 0.70 | 0.41 |
| <3-4 times/week | 363 | 98 | 0.54 (0.17-1.77) |  |  |
| 3-4 times/week | 181 | 50 | 0.51 (0.15-1.70) |  |  |
| >3-4 times/week | 120 | 33 | 0.50 (0.15-1.66) |  |  |
| Cream or sour cream |  |  |  |  |  |
| Never | 134 | 25 | Ref. | 0.52 | 0.22 |
| <1-3 times/month | 166 | 45 | 0.75 (0.44-1.27) |  |  |
| 1-3 times/month | 196 | 54 | 0.69 (0.41-1.15) |  |  |
| >1-3 time/month | 197 | 56 | 0.72 (0.43-1.19) |  |  |
| Ice-cream |  |  |  |  |  |
| Never | 52 | 14 | Ref. | 0.29 | 0.66 |
| <1-3 times/month | 162 | 40 | 0.83 (0.43-1.59) |  |  |
| 1-3 times/month – 1 time/week | 288 | 89 | 1.08 (0.52-2.21) |  |  |
| >1 time/week | 194 | 40 | 1.24 (0.61-2.49) |  |  |
| Yogurt |  |  |  |  |  |
| Never | 112 | 33 | Ref. | 0.18 | 0.34 |
| <2 times/week | 210 | 57 | 1.21 (0.74-1.98) |  |  |
| 2 – 3-4 times/week | 234 | 46 | 1.74 (0.91-2.99) |  |  |
| >3-4 times/week | 139 | 43 | 1.17 (0.69-1.98) |  |  |

^a^ All cases and all spouse controls using unconditional logistic regression models

^b^ Multivariable model: adjusted for age, sex, ethnicity, state and smoking status

**Supplementary Table 3c: Odds ratios and 95% confidence intervals for the likelihood of FL in relation to meat consumed in the year before last among cases and sibling controls (no imputation)**

| Exposures | Cases ^a^ | Sibling controls ^a^ | OR (95% CI) ^b^ | P value | P trend |
| --- | --- | --- | --- | --- | --- |
| Daily quantity of meat intake (grams/day) | | | | |  |
| Beef or veal (not corned) | | |  | |  |
| >91.4 | 70 | 77 | Ref. | 0.54 | 0.58 |
| 91.4-3.10 | 69 | 98 | 0.70 (0.42-1.16) |  |  |
| <31.0 | 88 | 107 | 0.89 (0.52-1.52) |  |  |
| Never | 11 | 17 | 0.76 (0.28-2.07) |  |  |
| Chicken | | |  | |  |
| >69.9 | 69 | 94 | Ref. | 0.84 | 0.48 |
| 69.9-32.6 | 90 | 101 | 1.22 (0.74-2.01) |  |  |
| <32.6 | 70 | 89 | 1.18 (0.70-1.98) |  |  |
| Never | 9 | 10 | 1.45 (0.44-4.74) |  |  |
| Lamb |  |  |  |  |  |
| >39.3 | 71 | 99 | Ref. | 0.87 | 0.63 |
| 39.3-18.4 | 61 | 74 | 1.14 (0.65-1.99) |  |  |
| <18.4 | 83 | 96 | 1.25 (0.71-2.21) |  |  |
| Never | 21 | 23 | 1.26 (0.55-2.87) |  |  |
| Pork (not corned or pickled) | | |  | |  |
| >27.2 | 43 | 62 | Ref. | 0.25 | 0.24 |
| 27.2-12.3 | 61 | 76 | 1.45 (0.75-2.80) |  |  |
| <12.3 | 86 | 84 | 1.01 (0.70-2.02) |  |  |
| Never | 41 | 60 | 1.43 (0.66-3.09) |  |  |
| Sausages |  |  |  |  |  |
| >15.5 | 48 | 67 | Ref. | 0.52 | 0.22 |
| 15.5-7.8 | 69 | 92 | 1.45 (0.80-2.65) |  |  |
| <7.8 | 80 | 92 | 1.56 (0.81-3.01) |  |  |
| Never | 39 | 44 | 1.67 (0.78-3.57) |  |  |
| Processed meat (e.g., ham, corned beef, prosciutto, salami) | | | | |  |
| >17.1 | 48 | 75 | Ref. | 0.46 | 0.65 |
| 17.1-6.0 | 93 | 95 | 1.93 (0.89-3.38) |  |  |
| <6.0 | 77 | 97 | 1.64 (0.82-2.95) |  |  |
| Never | 17 | 32 | 0.89 (0.37-2.13) |  |  |
| Bacon |  |  |  |  |  |
| >6.9 | 63 | 84 | Ref. | 0.34 | 0.25 |
| 6.9-3.4 | 64 | 84 | 1.68 (0.93-3.03) |  |  |
| <3.4 | 81 | 96 | 1.54 (0.86-2.75) |  |  |
| Never | 31 | 35 | 1.47 (0.66-3.28) |  |  |
| Total daily quantity of any meat intake (grams/day) ^c^ | | | | |  |
| >248.0 | 74 | 96 | Ref. | 0.89 | 0.57 |
| 248.0-120.0 | 82 | 98 | 1.20 (0.73-1.96) |  |  |
| <120.0 | 78 | 97 | 1.19 (0.68-2.07) |  |  |
| Never | 6 | 5 | 1.09 (0.29-4.05) |  |  |

^a^ Cases and their matched related controls using conditional logistic regression models

^b^ Multivariable model: adjusted for age, sex, ethnicity, state and smoking status

^c^ Total daily quantity of any meat intake was obtained by summing the daily grams per day of each type of meat

**Supplementary Table 3d: Odds ratios and 95% confidence intervals for the likelihood of FL in relation to meat consumed in the year before last among cases and spouse controls (no imputation)**

| Exposures | Cases ^a^ | Spouse controls ^a^ | OR (95% CI) ^b^ | P value | P trend |
| --- | --- | --- | --- | --- | --- |
| Daily quantity of meat intake (grams/day) | | | | |  |
| Beef or veal (not corned) | | |  | |  |
| >61.0 | 222 | 49 | Ref. | 0.18 | 0.64 |
| 61.0-30.5 | 187 | 64 | 1.63 (0.40-1.99) |  |  |
| <30.5 | 249 | 58 | 1.01 (0.65-1.59) |  |  |
| Never | 38 | 10 | 1.04 (0.47-2.33) |  |  |
| Chicken | | |  | |  |
| >58.4 | 227 | 60 | Ref. | 0.72 | 0.33 |
| 58.4-32.7 | 224 | 58 | 1.13 (0.74-1.71) |  |  |
| <32.7 | 220 | 56 | 1.29 (0.83-2.00) |  |  |
| Never | 19 | 6 | 1.02 (0.37-2.76) |  |  |
| Lamb |  |  |  |  |  |
| >39.3 | 208 | 51 | Ref. | 0.38 | 0.31 |
| 39.3-18.4 | 181 | 56 | 0.81 (0.51-1.28) |  |  |
| <18.4 | 236 | 62 | 1.06 (0.69-1.61) |  |  |
| Never | 57 | 11 | 1.44 (0.70-2.98) |  |  |
| Pork (not corned or pickled) | | |  | |  |
| >27.2 | 142 | 46 | Ref. | 0.17 | 0.18 |
| 27.2-12.3 | 187 | 50 | 1.33 (0.81-2.19) |  |  |
| <12.3 | 210 | 53 | 1.64 (0.90-2.68) |  |  |
| Never | 134 | 31 | 1.63 (0.94-2.69) |  |  |
| Sausages |  |  |  |  |  |
| >15.5 | 175 | 53 | Ref. | 0.11 | 0.30 |
| 15.5-7.8 | 200 | 48 | 1.52 (0.84-2.47) |  |  |
| <7.8 | 220 | 58 | 1.61 (0.82-2.55) |  |  |
| Never | 94 | 23 | 1.81 (0.72-3.21) |  |  |
| Processed meat (e.g., ham, corned beef, prosciutto, salami) | | | | |  |
| >17.1 | 173 | 47 | Ref. | 0.13 | 0.22 |
| 17.1-6.0 | 208 | 61 | 1.05 (0.66-1.66) |  |  |
| <6.0 | 238 | 61 | 1.30 (0.85-2.01) |  |  |
| Never | 69 | 12 | 0.56 (0.35-1.05) |  |  |
| Bacon |  |  |  |  |  |
| >6.9 | 198 | 56 | Ref. | 0.15 | 0.80 |
| 6.9-3.4 | 188 | 49 | 1.24 (0.78-1.98) |  |  |
| <3.4 | 227 | 63 | 1.32 (0.87-2.02) |  |  |
| Never | 84 | 12 | 0.72 (0.33-1.30) |  |  |
| Total daily quantity of any meat intake (grams/day) ^c^ | | | | |  |
| >230.0 | 257 | 58 | Ref. | 0.21 | 0.57 |
| 230.0-127.6 | 194 | 64 | 0.74 (0.47-1.16) |  |  |
| <127.6 | 234 | 56 | 1.15 (0.74-1.78) |  |  |
| Never | 14 | 8 | 0.80 (0.26-2.43) |  |  |

^a^ All cases and all spouse controls using unconditional logistic regression models

^b^ Multivariable model: adjusted for age, sex, ethnicity, state and smoking status

^c^ Total daily quantity of any meat intake was obtained by summing the daily grams per day of each type of meat

**Supplementary Table 3e: Odds ratios and 95% confidence intervals for the likelihood of FL in relation to fish and seafood consumed in the year before last among cases and sibling controls (no imputation)**

| Exposures | Cases ^a^ | Sibling controls ^a^ | OR (95% CI) ^b^ | P value | P trend |
| --- | --- | --- | --- | --- | --- |
| Daily quantity of fish and seafood intake (grams/day) | | | | |  |
| Oily fish | | |  | |  |
| Never | 45 | 83 | Ref. | 0.04 | 0.02 |
| <8.6 | 80 | 72 | 1.69 (0.88-3.24) |  |  |
| 8.6-12.9 | 57 | 72 | 1.80 (0.92-3.51) |  |  |
| >12.9 | 55 | 61 | 2.09 (1.18-3.68) |  |  |
| Tuna | | |  | |  |
| Never | 54 | 63 | Ref. | 0.58 | 0.43 |
| <6.0 | 59 | 85 | 0.78 (0.42-1.45) |  |  |
| 6.1-12.0 | 49 | 60 | 1.15 (0.60-2.23) |  |  |
| >12.0 | 72 | 80 | 1.20 (0.62-2.30) |  |  |
| White fish |  |  |  |  |  |
| Never | 14 | 27 | Ref. | 0.21 | 0.08 |
| <18.4 | 70 | 98 | 1.88 (0.79-4.50) |  |  |
| 18.4-39.3 | 81 | 97 | 1.86 (0.81-4.73) |  |  |
| >39.3 | 72 | 71 | 2.53 (0.95-6.61) |  |  |
| Crustaceans | | |  | |  |
| Never | 68 | 85 | Ref. | 0.94 | 0.76 |
| <4.5 | 51 | 75 | 0.97 (0.51-1.84) |  |  |
| 4.5-9.0 | 59 | 69 | 1.04 (0.57-1.89) |  |  |
| >9.0 | 47 | 50 | 0.84 (0.40-1.75) |  |  |
| Shellfish |  |  |  |  |  |
| Never | 103 | 120 | Ref. | 0.10 | 0.21 |
| <3.0 | 59 | 56 | 1.60 (0.86-2.96) |  |  |
| 3.0-6.0 | 41 | 51 | 1.05 (0.55-2.00) |  |  |
| >6.0 | 27 | 52 | 0.52 (0.34-1.15) |  |  |
| Other seafood | | | | |  |
| Never | 84 | 110 | Ref. | 0.95 | 0.93 |
| <6.0 | 49 | 53 | 1.10 (0.56-2.16) |  |  |
| 6.0-17.1 | 39 | 49 | 0.91 (0.45-1.86) |  |  |
| >17.1 | 39 | 40 | 1.11 (0.53-2.30) |  |  |
| Total daily quantity of fish or seafood intake (grams/day) ^c^ | | | | |  |
| <26.2 | 60 | 98 | Ref. | 0.63 | 0.22 |
| 26.2-51.5 | 95 | 96 | 1.25 (0.29-5.51) |  |  |
| >51.5 | 82 | 96 | 2.44 (0.58-10.31) |  |  |
| Never | 4 | 11 | 2.43 (0.56-10.44) |  |  |

^a^ Cases and their matched related controls using conditional logistic regression models

^b^ Multivariable model: adjusted for age, sex, ethnicity, state and smoking status

^c^ Total daily quantity of fish or seafood intake was obtained by summing the daily grams per day of each type of fish or seafood

**Supplementary Table 3f: Odds ratios and 95% confidence intervals for the likelihood of FL in relation to fish and seafood consumed in the year before last among cases and spouse controls (no imputation)**

| Exposures | Cases ^a^ | Spouse controls ^a^ | OR (95% CI) ^b^ | P value | P trend |
| --- | --- | --- | --- | --- | --- |
| Daily quantity of fish and seafood intake (grams/day) | | | | |  |
| Oily fish | | |  | |  |
| Never | 132 | 33 | Ref. | 0.95 | 0.56 |
| <7.5 | 193 | 51 | 0.95 (0.58-1.55) |  |  |
| 7.5-12.9 | 192 | 51 | 0.92 (0.57-1.49) |  |  |
| >12.9 | 166 | 47 | 0.87 (0.52-1.42) |  |  |
| Tuna | | |  | |  |
| Never | 157 | 34 | Ref. | 0.28 | 0.49 |
| <8.6 | 193 | 51 | 0.73 (0.46-1.17) |  |  |
| 8.6-12.0 | 116 | 46 | 0.49 (0.30-1.18) |  |  |
| >12.0 | 211 | 48 | 0.90 (0.55-1.45) |  |  |
| White fish |  |  |  |  |  |
| Never | 42 | 12 | Ref. | 0.63 | 0.95 |
| <9.0 | 200 | 58 | 0.97 (0.45-2.14) |  |  |
| 9.0-15.0 | 228 | 62 | 0.98 (0.46-2.12) |  |  |
| >15.0 | 214 | 52 | 1.09 (0.50-2.38) |  |  |
| Crustaceans | | |  | |  |
| Never | 180 | 43 | Ref. | 0.24 | 0.48 |
| <4.5 | 138 | 47 | 0.61 (0.38-1.12) |  |  |
| 4.5-9.0 | 185 | 46 | 0.76 (0.47-1.24) |  |  |
| >9.0 | 154 | 38 | 0.83 (0.52-1.32) |  |  |
| Shellfish |  |  |  |  |  |
| Never | 286 | 75 | Ref. | 0.70 | 0.35 |
| <3.0 | 144 | 40 | 0.80 (0.52-1.23) |  |  |
| 3.0-6.0 | 123 | 31 | 0.86 (0.54-1.38) |  |  |
| >6.0 | 109 | 32 | 0.81 (0.51-1.31) |  |  |
| Other seafood | | | | |  |
| Never | 273 | 75 | Ref. | 0.68 | 0.53 |
| <4.5 | 119 | 33 | 0.90 (0.56-1.41) |  |  |
| 4.5-6.0 | 100 | 31 | 0.73 (0.44-1.21) |  |  |
| >6.0 | 129 | 31 | 0.95 (0.59-1.53) |  |  |
| Total daily quantity of fish or seafood intake (grams/day) ^c^ | | | | |  |
| <32.2 | 235 | 60 | Ref. | 0.93 | 0.96 |
| 32.2-53.2 | 216 | 60 | 1.14 (0.36-3.59) |  |  |
| >53.2 | 238 | 59 | 1.02 (0.32-3.20) |  |  |
| Never | 18 | 7 | 1.14 (0.36-3.57) |  |  |

^a^ All cases and all spouse controls using unconditional logistic regression models

^b^ Multivariable model: adjusted for age, sex, ethnicity, state and smoking status

^c^ Total daily quantity of fish or seafood intake was obtained by summing the daily grams per day of each type of fish or seafood

**Supplementary Table 4a: Odds ratios and 95% confidence intervals for the likelihood of FL in relation to diary food and fats consumed in the year before last among cases and their matched spouse controls**

| Exposures | Cases ^a^ | Spouse controls ^a^ | OR (95% CI) ^b^ | P value |
| --- | --- | --- | --- | --- |
| Margarine type ^c^ | | | | |
| None | 39 | 39 | Ref. | 0.85 |
| Animal-based only | 32 | 27 | 1.63 (0.60-4.50) |  |
| Plant-based only | 89 | 90 | 1.14 (0.49-2.63) |  |
| Animal- and plant-based | 23 | 26 | 1.17 (0.42-3.22) |  |
| Cow milk type ^b^ | | | | |
| None | 11 | 16 | Ref. | 0.11 |
| Full cream only | 45 | 46 | 1.30 (0.10-2.53) |  |
| Low fat (≤3% fat) only | 113 | 108 | 1.50 (0.30-2.71) |  |
| Full and low fat | 17 | 15 | 1.54 (0.11-2.97) |  |
| Quantity of cow milk per day ^b^ | | | | |
| Never | 11 | 16 | Ref. | 0.10 |
| <250 ml | 49 | 50 | 1.18 (0.59-3.91) |  |
| 250 ml | 66 | 67 | 1.16 (0.48-3.89) |  |
| ≥500 ml | 60 | 52 | 1.39 (0.11-3.71) |  |
| Frequency of diary and fat consumption ^c^ | | | | |
| Margarine on cooked vegetables | | | | |
| Never | 108 | 106 | Ref. | 0.83 |
| <1 time/week | 29 | 36 | 0.91 (0.45-1.84) |  |
| ≥1 time/week | 44 | 38 | 1.14 (0.57-2.27) |  |
| Butter/dairy blend on cooked vegetables | | | | |
| Never | 108 | 97 | Ref. | 0.49 |
| <1 time/week | 32 | 39 | 0.73 (0.33-1.64) |  |
| ≥1 time/week | 43 | 42 | 1.13 (0.55-2.35) |  |
| Mayonnaise |  |  |  |  |
| Never | 43 | 39 | Ref. | 0.62 |
| <1 time/week | 93 | 86 | 1.13 (0.70-2.44) |  |
| ≥1 time/week | 49 | 55 | 1.21 (0.55-2.64) |  |
| Ricotta or cottage cheese | | | | |
| Never | 88 | 84 | Ref. | 0.12 |
| <1 time/month | 46 | 42 | 0.99 (0.52-1.91) |  |
| ≥1 time/month | 45 | 55 | 0.58 (0.29-1.18) |  |
| All other cheeses | | | | |
| Never | 14 | 3 | Ref. | 0.34 |
| ≤2 times/week | 88 | 98 | 0.31 (0.06-1.72) |  |
| >2 times/week | 81 | 83 | 0.37 (0.07-2.02) |  |
| Cream or sour cream |  |  |  |  |
| Never | 30 | 25 | Ref. | 0.38 |
| <1 time/week | 91 | 99 | 0.96 (0.39-2.34) |  |
| ≥1 time/week | 61 | 56 | 1.45 (0.57-3.72) |  |
| Ice-cream |  |  |  |  |
| Never | 12 | 14 | Ref. | 0.36 |
| <1 time/week | 88 | 93 | 1.72 (0.60-5.00) |  |
| ≥1 time/week | 83 | 76 | 2.02 (0.67-6.12) |  |
| Yogurt |  |  |  |  |
| Never | 31 | 33 | Ref. | 0.20 |
| ≤2 times/week | 79 | 82 | 1.43 (0.67-3.06) |  |
| >2 times/week | 75 | 64 | 2.02 (0.95-4.29) |  |

^a^ Cases and their matched unrelated controls using conditional logistic regression

^b^ Multivariable model – adjusted for age, sex, ethnicity, state and smoking status

^c^ Imputations (number of participants with missing values): margarine type consumed (7), cow milk type consumed (1), quantity of cow milk consumed (1), frequency of diary and fat consumption (11)

**Supplementary Table 4b: Odds ratios and 95% confidence intervals for the likelihood of FL in relation to meat consumed in the year before last among cases and their matched spouse controls**

| Exposures | Cases ^a^ | Spouse controls ^a^ | OR (95% CI) ^b^ | P value | P trend |
| --- | --- | --- | --- | --- | --- |
| Daily quantity of meat intake (grams/day) ^c^ | | | | |  |
| Beef or veal (not corned) | | |  | |  |
| >61.0 | 65 | 49 | Ref. | 0.13 | 0.76 |
| 61.0-30.5 | 43 | 64 | 0.54 (0.28-1.02) |  |  |
| <30.5 | 61 | 58 | 1.02 (0.51-2.05) |  |  |
| Never | 10 | 10 | 1.25 (0.36-4.35) |  |  |
| Chicken | | |  | |  |
| >58.4 | 51 | 60 | Ref. | 0.53 | 0.31 |
| 58.4-32.7 | 45 | 58 | 1.19 (0.60-2.38) |  |  |
| <32.7 | 77 | 56 | 1.36 (0.83-3.31) |  |  |
| Never | 3 | 6 | 1.53 (0.34-7.79) |  |  |
| Lamb |  |  |  |  |  |
| >39.3 | 49 | 51 | Ref. | 0.29 | 0.14 |
| 39.3-18.4 | 48 | 56 | 1.00 (0.50-1.98) |  |  |
| <18.4 | 68 | 62 | 1.84 (0.86-3.95) |  |  |
| Never | 12 | 11 | 1.52 (0.40-5.75) |  |  |
| Pork (not corned or pickled) | | |  | |  |
| >27.2 | 34 | 46 | Ref. | 0.33 | 0.13 |
| 27.2-12.3 | 53 | 50 | 1.51 (0.73-3.10) |  |  |
| <12.3 | 56 | 53 | 2.09 (0.96-4.60) |  |  |
| Never | 33 | 31 | 1.77 (0.57-5.52) |  |  |
| Sausages |  |  |  |  |  |
| >15.5 | 42 | 53 | Ref. | 0.31 | 0.12 |
| 15.5-7.8 | 56 | 48 | 1.72 (0.87-3.38) |  |  |
| <7.8 | 58 | 58 | 2.08 (0.92-4.67) |  |  |
| Never | 20 | 23 | 1.98 (0.67-5.91) |  |  |
| Processed meat (e.g., ham, corned beef, prosciutto, salami) | | | | |  |
| >17.1 | 43 | 47 | Ref. | 0.32 | 0.60 |
| 17.1-6.0 | 45 | 61 | 1.19 (0.54-2.61) |  |  |
| <6.0 | 70 | 61 | 2.75 (0.81-6.82) |  |  |
| Never | 16 | 12 | 2.73 (0.50-7.25) |  |  |
| Bacon |  |  |  |  |  |
| >6.9 | 50 | 56 | Ref. | 0.17 | 0.29 |
| 6.9-3.4 | 49 | 49 | 1.53 (0.78-3.01) |  |  |
| <3.4 | 59 | 63 | 1.97 (0.88-4.41) |  |  |
| Never | 20 | 12 | 3.54 (0.31-7.27) |  |  |
| Total daily quantity of any meat intake (grams/day) ^c, d^ | | | | |  |
| >230.0 | 67 | 58 | Ref. | 0.44 | 0.52 |
| 230.0-127.6 | 45 | 64 | 0.56 (0.29-1.07) |  |  |
| <127.6 | 66 | 56 | 1.49 (0.73-3.09) |  |  |
| Never | 3 | 8 | 2.71 (0.23-10.07) |  |  |

^a^ Cases and their matched unrelated controls using conditional logistic regression

^b^ Multivariable model – adjusted for age, sex, ethnicity, state and smoking status

^c^ Imputations (number of participants with missing values): daily quantity of meat intake (11), daily quantity of any meat intake (2)

^d^ Categories of meat plate portion size: small (<B), medium (B-<C), large (≥C) – see appendix 1

**Supplementary Table 4c: Odds ratios and 95% confidence intervals for FL risk in relation to fish or seafood consumed in the year before last among cases and their matched spouse controls**

| Exposures | Cases ^a^ | Spouse controls ^a^ | OR (95% CI) ^b^ | P value | P trend |
| --- | --- | --- | --- | --- | --- |
| Daily quantity of fish and seafood intake (grams/day) ^c^ | | | | |  |
| Oily fish | | |  | |  |
| Never | 37 | 33 | Ref. | 0.59 | 0.62 |
| <7.5 | 51 | 51 | 0.52 (0.21-1.32) |  |  |
| 7.5-12.9 | 48 | 51 | 0.60 (0.23-1.54) |  |  |
| >12.9 | 46 | 47 | 0.64 (0.23-1.77) |  |  |
| Tuna | | |  | |  |
| Never | 44 | 34 | Ref. | 0.36 | 0.91 |
| <8.6 | 51 | 51 | 0.49 (0.19-1.26) |  |  |
| 8.6-12.0 | 44 | 46 | 0.30 (0.11-1.18) |  |  |
| >12.0 | 45 | 48 | 0.81 (0.31-2.08) |  |  |
| White fish |  |  |  |  |  |
| Never | 10 | 12 | Ref. | 0.41 | 0.25 |
| <9.0 | 49 | 58 | 2.77 (0.71-10.73) |  |  |
| 9.0-15.0 | 60 | 62 | 3.28 (0.80-13.45) |  |  |
| >15.0 | 58 | 52 | 3.28 (0.81-13.36) |  |  |
| Crustaceans | | |  | |  |
| Never | 48 | 43 | Ref. | 0.63 | 0.19 |
| <4.5 | 43 | 47 | 0.82 (0.37-1.83) |  |  |
| 4.5-9.0 | 41 | 46 | 0.66 (0.26-1.65) |  |  |
| >9.0 | 35 | 38 | 0.52 (0.19-1.43) |  |  |
| Shellfish |  |  |  |  |  |
| Never | 77 | 75 | Ref. | 0.69 | 0.93 |
| <3.0 | 33 | 40 | 0.68 (0.31-1.49) |  |  |
| 3.0-6.0 | 35 | 31 | 1.08 (0.51-2.31) |  |  |
| >6.0 | 25 | 32 | 0.94 (0.40-2.27) |  |  |
| Other seafood | | | | |  |
| Never | 77 | 75 | Ref. | 0.60 | 0.34 |
| <4.5 | 28 | 33 | 0.62 (0.28-1.37) |  |  |
| 4.5-6.0 | 27 | 31 | 0.63 (0.27-1.43) |  |  |
| >6.0 | 29 | 31 | 0.67 (0.28-1.62) |  |  |
| Total daily quantity of fish or seafood intake (grams/day) ^d^ | | | | |  |
| <32.2 | 55 | 60 | Ref. | 0.92 | 0.98 |
| 32.2-53.2 | 59 | 60 | 2.71 (0.55-13.20) |  |  |
| >53.2 | 62 | 59 | 2.49 (0.48-12.79) |  |  |
| Never | 4 | 7 | 2.88 (0.53-13.54) |  |  |

^a^ Cases and their matched unrelated controls using conditional logistic regression

^b^ Multivariable model – adjusted for age, sex, ethnicity, state and smoking status

^c^ Imputations (number of participants with missing values): daily quantity of fish or seafood intake (35)

^d^ Total daily quantity of fish or seafood intake was obtained by summing the daily grams per day of each type of fish or seafood

**APPENDIX**

1. Meat plate portion size


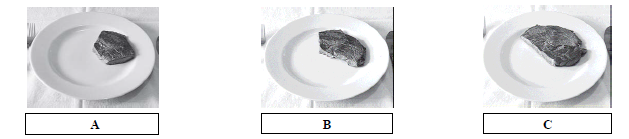


1. Meat average portion size factor used to scale participants food intake

| Meat portion sizes | Average portion size factor |
| --- | --- |
| Don’t eat meat | 0 |
| Less than A | 0.4 |
| A | 0.5 |
| Between A & B | 0.75 |
| B (Average portion) | 1 |
| Between B & C | 1.5 |
| C (Double portion) | 2 |
| More than C | 2.5 |

1. Standard meat serving sizes – from Melbourne Collaborative Cancer Study dietary recall (16)

| Food item | Portion size (grams) |
| --- | --- |
| Bacon | 45.77 |
| Beef | 213.2 |
| Chicken | 163.23 |
| Lamb | 183.32 |
| Pork | 163.6 |
| Processed meat | 59.91 |
| Sausages | 103.51 |

1. Daily equivalent frequency of intake per day

| Frequency of intake | Daily equivalent |
| --- | --- |
| None | 0 |
| Less than once per month | 0.02 |
| 1-3 times per month | 0.07 |
| 1 time per week | 0.14 |
| 2 times per week | 0.28 |
| 3-4 times per week | 0.5 |
| 5-6 times per week | 0.78 |
| 1 time a day | 1 |
| 2 times a day | 2 |
| 3 or more times a day | 3 |

**References**

1. Schecter A, Colacino J, Haffner D, Patel K, Opel M, Päpke O*, et al.* Perfluorinated compounds, polychlorinated biphenyls, and organochlorine pesticide contamination in composite food samples from Dallas, Texas, USA. Environ Health Perspect. 2010;118(6):796-802.

2. Odutola MK, Nnakelu E, Giles GG, van Leeuwen MT, Vajdic CM. Lifestyle and risk of follicular lymphoma: a systematic review and meta-analysis of observational studies. Cancer Causes Control. 2020;31(11):979-1000.

3. Mu M, Xu LF, Hu D, Wu J, Bai MJ. Dietary patterns and overweight/obesity: a review article. Iran J Public Health. 2017;46(7):869-76.

4. Chiolero A, Jacot-Sadowski I, Faeh D, Paccaud F, Cornuz J. Association of cigarettes smoked daily with obesity in a general adult population. Obesity. 2007;15(5):1311-8.

5. Carreras-Torres R, Johansson M, Haycock PC, Relton CL, Davey Smith G, Brennan P*, et al.* Role of obesity in smoking behaviour: Mendelian randomisation study in UK Biobank. BMJ. 2018;361:k1767.

6. MacLean RR, Cowan A, Vernarelli JA. More to gain: dietary energy density is related to smoking status in US adults. BMC Public Health. 2018;18(1):365.

7. Molarius A, Seidell JC, Kuulasmaa K, Dobson AJ, Sans S. Smoking and relative body weight: an international perspective from the WHO MONICA Project. J Epidemiol Community Health. 1997;51(3):252-60.

8. Passarelli MN, Newcomb PA, Hampton JM, Trentham-Dietz A, Titus LJ, Egan KM*, et al.* Cigarette smoking before and after breast cancer diagnosis: mortality from breast cancer and smoking-related diseases. J Clin Oncol. 2016;34(12):1315-22.

9. Hales CM, Carroll MD, Fryar CD, Ogden CL. Prevalence of obesity and severe obesity among adults: United States, 2017-2018. NCHS Data Brief. 2020(360):1-8.

10. Bista A, Sharma S, Shah BK. Disparities in receipt of radiotherapy and survival by age, sex, and ethnicity among patient with stage I follicular lymphoma. Front Oncol. 2016;6:101-01.

11. Geyer SM, Morton LM, Habermann TM, Allmer C, Davis S, Cozen W*, et al.* Smoking, alcohol use, obesity, and overall survival from non-Hodgkin lymphoma: a population-based study. Cancer. 2010;116(12):2993-3000.

12. Han X, Stevens J, Bradshaw PT. Body mass index, weight change, and survival in non-Hodgkin lymphoma patients in Connecticut women. Nutr Cancer. 2013;65(1):43-50.

13. Foerster B, Pozo C, Abufaraj M, Mari A, Kimura S, D’Andrea D*, et al.* Association of smoking status with recurrence, metastasis, and mortality among patients with localized prostate cancer undergoing prostatectomy or radiotherapy: a systematic review and meta-analysis. JAMA Oncology. 2018;4(7):953-61.

14. Peters SA, Huxley RR, Woodward M. Do smoking habits differ between women and men in contemporary Western populations? Evidence from half a million people in the UK Biobank study. BMJ Open. 2014;4(12):e005663.

15. El-Toukhy S, Sabado M, Choi K. Trends in susceptibility to smoking by race and ethnicity. Pediatrics. 2016;138(5).

16. Bassett JK, English DR, Fahey MT, Forbes AB, Gurrin LC, Simpson JA*, et al.* Validity and calibration of the FFQ used in the Melbourne Collaborative Cohort Study. Public Health Nutr. 2016;19(13):2357-68.
